# Infant crying and the calming response: Parental versus mechanical soothing using swaddling, sound, and movement

**DOI:** 10.1371/journal.pone.0214548

**Published:** 2019-04-24

**Authors:** Eline L. Möller, Wieke de Vente, Roos Rodenburg

**Affiliations:** 1 Stichting Epilepsie Instellingen Nederland, Heemstede, The Netherlands; 2 Research Institute of Child Development and Education, University of Amsterdam, Amsterdam, The Netherlands; 3 Research Priority Area Yield, University of Amsterdam, Amsterdam, The Netherlands; Utrecht University, NETHERLANDS

## Abstract

**Background:**

Frequent infant crying is associated with parental exhaustion, depression, or even infant hospitalization and shaken baby syndrome. Effective prompt soothing methods are lacking for infants under 6 months. We examined whether swaddling, sound, and movement evoked an immediate calming response (CR) when parents soothed their infants and using a smart crib, and whether infant age affected the CR.

**Methods:**

Infants’ CR was assessed in a community sample of 69 infants (0–6 months) in a counterbalanced experiment with two conditions (parent, smart crib) each composed of three two-minute phases (baseline, supine, soothing). During baseline 1, parent and infant were sitting together; in supine 1, fussiness was elicited by putting the infant suddenly supine, followed by parental soothing (shushing and jiggling of the swaddled infant). Baseline 2, supine 2, and soothing by the crib followed. Fussiness was observed and infant heart rate (HR) and heart rate variability (HRV) were recorded. The CR was operationalized as decreased fussiness and HR, and increased HRV during soothing compared to lying supine.

**Results:**

Infant fussiness and HR were lower in both soothing phases compared to the supine phases. Infant HRV tended to be higher during parental soothing than during supine, but did not significantly differ between mechanical soothing and supine. Younger infants responded with a stronger CR (decreased fussiness and increased HRV) to parental soothing, but not to mechanical soothing. For HR, infants’ CR was stronger in the crib than in the parent condition, whereas for HRV, infants’ CR was stronger in the parent condition. For fussiness, infants’ CR tended to be stronger in the parent condition.

**Conclusion:**

Parental and mechanical soothing using swaddling, sound, and movement promptly induced a CR in infants. This has important clinical implications for soothing fussy and crying infants. Future studies should investigate the effects of parental versus mechanical soothing in the home setting.

## Introduction

Crying is part of normal infant behavior and plays an important role in the mutual regulation between infant and parent. However, up to 20% of infants cry excessively [1]. Wessel’s definition of infant excessive crying is often used: crying for at least three hours per day for at least three days per week, and at least for three weeks in a row [2]. In clinical practice, this definition is not very useful as parents may also perceive less frequent crying as a problem. Crying is therefore considered excessive when parents experience it as such [3].

Crying problems are burdening to infants and parents and are associated among others with impaired infant sleep [4], parental exhaustion [5] and depression [6], and shaken baby syndrome [7]. For some families, the excessive crying is so unbearable that infants are hospitalized to alleviate parental stress and family disturbance [8]. Only in 5% of excessively crying infants, however, a medical cause for the crying can be found [1]. Effects of behavioral methods to reduce infant cry and fuss problems in infants younger than six months have rarely been reported and do not take into account the immaturity of newborn babies [9]. New solutions that reduce infant crying are therefore warranted.

Such a possible solution is the Happiest Baby method (HB) [10]. According to Karp [10] recreating the sensory milieu of the womb (e.g., snug position, floating in fetus position, deep resonant sound of the placental blood flow, jiggling motion, swallowing of amniotic fluid) would calm infants by triggering infants’ so called calming response (CR). In HB infants’ CR is triggered via a bundle of 5 stimuli or steps (5S’s) the moment the baby is crying: (1) swaddling; (2) side/stomach position in the arms of the parent; (3) shushing; (4) swinging; and (5) sucking. Each of these 5S’s has a calming effect on infants [11–16]. Infants who were simultaneously soothed with the 5S’s by a researcher after immunization showed decreased duration of crying and lower mean pain scores compared to infants who were given either water or sucrose pre-vaccination and were soothed as usual post-vaccination [17]. HB also appears to significantly decrease infants’ excessive crying in infants under 4 months of age [18].

HB, however, requires the availability of the parent to soothe the infant, but parents also need rest, for example during the night. Following Kurth’s system model on infant crying and maternal fatigue [5], infants who are easily soothed allow their parents to recover. Well-rested parents are better able to take care of their infants and to help them regulate themselves, resulting in more successful soothing and positive parent-child interactions. On the contrary, parents who have enduring difficulties with soothing their infant do not get enough rest. As a consequence, it is more difficult for exhausted parents to exhibit adequate parenting behaviors and to calm the infant, causing the baby to cry even more. Parent and infant may then end up in a vicious circle, in which the baby and the parent bring each other out of balance time after time. A solution to break this vicious circle or to prevent parental exhaustion may lie in mechanical soothing. Recently, a smart crib has been developed that makes use of three of HB’s 5S’s (swaddling, shushing via white noise, and swinging) to calm infants [19]. If mechanical soothing using swaddling, sound, and movement is as effective as parental soothing using the same stimuli, mechanical soothing may be used during periods in which the exhausted parent needs rest, such as during the night.

Infant’s age might influence the strength of the CR. According to Karp [10], infants would actually need a fourth trimester in the womb to mature, which is biologically impossible. In the first three months after birth, infants would still be very sensitive to these intra-uterine stimulations, after which the CR would gradually diminish. By then infants would be able to regulate themselves better and are more ready for the requirements of the extra-uterine environment. This fits with evidence that infants experience the first biobehavioral shift around 3 months, during which the behavior and physiology of infants shifts from intra-uterine to more extra-uterine regulation [20–23]. Around this period, several changes in infants’ behavioral development can be observed [22]: a more diurnal sleep/wake cycle appears with longer periods of consolidated sleep during the night, infants show enhanced habituation and classical and operant conditioning, and begin to show more responsive socially-oriented behavior, such as eye-contact, smiling, and cooing. On a more physiological level, infants are also more able to regulate themselves and become less dependent on their caregivers. For example, Van Puyvelde et al. [23] found that infants adjusted their respiratory sinus arrhythmia (RSA) levels to their mothers’ RSA levels in the first two months of life, but this relationship disappeared at three months of age. Thus, offering calming sensory stimuli for soothing might be more important for younger than for older infants.

To date, no study has compared parental elicitation of the CR with a mechanical device that elicits such a response nor investigated whether infant age affects the strength of the CR. Obtaining more insight in offering sensory stimuli by different “providers” (i.e., parent or device) for soothing would enable us not only to investigate whether human contact is needed to elicit a CR, but would also enable (infant) mental health professionals to fine-tune the guiding and treatment of both infants and parents for inconsolable crying. Most studies investigating the calming effects of sensory stimuli focused on a single stimulus, whereas we assessed infants’ CR to a combination of stimuli. We also adopted a multimethod approach by using behavioral observations and physiological measures to study the CR.

We examined infants’ CR to parental and mechanical soothing using a combination of swaddling, sound, and movement. The following questions were examined: (1) Do swaddling, sound, and movement by means of parental and mechanical soothing induce a CR in infants?; (2) Is the CR stronger in younger than in older infants?; and (3) Is there a difference in the strength of the calming response between parental and mechanical soothing? The strength of the CR was examined using behavioral observations of infant fussiness and physiological measures of arousal, that is heart rate (HR), which reflects the balance of the sympathetic and parasympathetic nervous system [24], and heart rate variability (HRV) in the high frequency domain, a relatively pure index of parasympathetic activity [25]. We hypothesized that a CR in response to swaddling, sound, and movement would be demonstrated by a significant decrease of observed infant fussiness, and a physiological pattern of relaxation, or in other words, a shift towards less sympathetic and more parasympathetic activation, as reflected in a significant decrease of HR, and a significant increase in HRV, as compared to induced distress. In addition, we expected that the younger the age of the infant, the stronger the CR of the infant. As in both soothing conditions swaddling, sound, and movement were used, no differences in the strength of the CR were expected between parental and mechanical soothing.

## Materials and methods

### Participants

**Fig 1:**
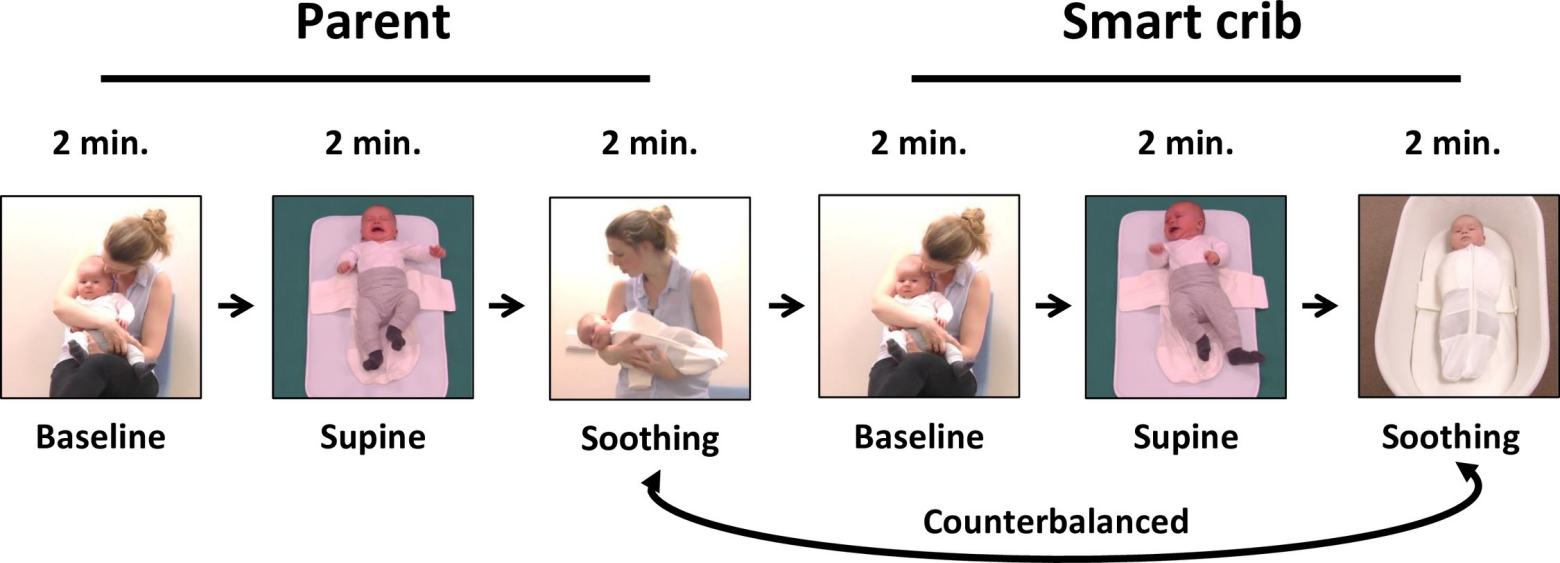
Overview of the experiment: Two conditions (smart crib/parent) with three phases (baseline, supine, soothing). The individual in this figure has given written informed consent (as outlined in PLOS consent form) to publish these case details.

Participants were 69 infants (37 boys) and one of their parents (67 mothers). Gestational age of the infants was on average 39.58 weeks (*SD* = 1.78, range 30.29−41.86 weeks). Three infants were born with a gestational age below 37 weeks. For these infants, corrected age was calculated using the chronologic age and adjusting for gestational age, that is, for the number of additional weeks from term (37 weeks). The (corrected) mean age of the infants was 13.85 weeks (*SD* = 6.57, range = 3.99−27.19 weeks). Parents were on average 33.43 years (*SD* = 3.63, range = 26.40−42.50 years). Almost all parents were married/cohabitating (*n* = 66) and born in the Netherlands (*n* = 62). Parents had a relatively high educational level (*M* = 6.64, *SD* = .64, on a scale from 1 (primary school) to 7 (university)). Participants were recruited through leaflets provided by child care centers, midwives, general practitioners, and the obstetrics department of two hospitals in Amsterdam, and via websites and Facebook groups that parents visit often. Parents were asked to participate in a study on the calming effects of swaddling, movement, and sound on infants. Parents received information about the study beforehand and had to sign informed consent. The study was approved by the ethical committee of the Research Institute of Child Development and Education of the University of Amsterdam (number 2017-CDE-7556). The individual depicted in Fig 1 has given written informed consent (as outlined in PLOS consent form) to publish these case details.

### Procedure

Parents could sign themselves up for our study via our website. Parents that wanted to participate were contacted by phone to explain the study and to make an appointment for a visit to our laboratory, and received a detailed information letter by email. Lab visits took place on different times during the day and were scheduled after a nap of the infant. At the lab visit, parents and infants were generally at ease. The experimenter first took the parent and infant to our waiting room, which looks like a cozy living room, and made sure the parent and infant were feeling comfortable before going to our lab room for the experiment. Parents were asked to make sure that their infant was not hungry or needing a diaper change before starting the experiment. If the infant was hungry, parents first fed their child before the experiment was started. The experimenter first explained the procedure and parents signed an informed consent form. Then, the experimenter practiced the soothing (shushing and swinging) of the infant with the parent, to ensure that all parents soothed their infants in the same way. To prevent that the contact with the parent during the swaddling already calmed the infants, the infant was swaddled in a swaddle sack by the experimenter. After electrodes were attached to the infant for the physiological measures, the experimental task to measure infants’ CR started, which lasted 12 minutes (for a description of the task, see Measurements). The parent and the infant were filmed during the task with four video cameras for behavioral measures that were coded afterwards. At the end of the experiment, parents received a refund of travel expenses and were taught HB [10] as a thank you gift. Parents’ physiological data were also recorded during the experiment and parents completed a set of questionnaires, but these data are not reported in the present study.

### Measurements

#### Setting and procedure

Infants’ CR was assessed with an experimental task that consisted of two conditions (parent, smart crib) each composed of three phases (baseline, supine, soothing) (see Fig 1). Each phase lasted two minutes. During the baseline phase of the parent condition, the parent sat on a chair with the infant on his lap. The parent was allowed to quietly interact and instructed to move as little as possible. Then, the supine condition started. The experimenter took the infant over from the parent, and suddenly lowered her hands rapidly about 20 centimeter and then abruptly stopped, a procedure that usually elicits the Moro reflex [26]. The experimenter then immediately put the infant on his back on a mat on the floor two meters away from the parent. The parent was instructed to turn around and to refrain from any verbal and non-verbal contact with the infant. The infant then lied on his back for two minutes, while the experimenter, out of sight of the infant, kept an eye on the child. Both the eliciting of the Moro-reflex and/or the unavailability of the parent were expected to have a distressing effect on the infant. Then, the experimenter swaddled the infant in a swaddle sack, while she did not interact with the infant (i.e., making no eye contact and with a neutral facial expression). The parental soothing phase then started. The parent soothed the infant with the HB method [10]: the infant was handed over to the parent, and the parent held the child in a side position (back of the infant against the chest of the parent) and made a shushing sound close to the ear of the infant while swinging the infant (i.e., making small jiggly movements while supporting the head of the infant).

After the experimenter took the infant out of the swaddle sack and handed the infant to the parent, the baseline phase of the smart crib condition started. Again, the parent and the infant sat together on a chair for two minutes. Then the second supine phase started in which fussiness was again elicited. Thereafter, the infant was again swaddled in a swaddle sack. Lastly, during the smart crib soothing phase, the infant was placed supine in the crib and the swaddle sack was attached to the crib. The crib was put on the highest speed and sound level, moving horizontally 6 cm either side during 3.2 cycles per second, and with white noise playing on 84dB. The parent was again instructed to turn around and to make no contact with the baby. The experimenter again made sure the infant was ok while out of sight of the infant. Order of the smart crib condition and parent condition was counterbalanced across infants.

#### Behavioral coding of infant fussiness

Our coding scheme was based on two well-known and validated coding schemes: the Laboratory Temperament Assessment Battery (Lab-TAB Prelocomotor version) [27], and the AFFEX system for the coding of facial expressions [28].

Infant fussiness was observed in both supine and both soothing phases. Infant fussiness could not reliably be observed during both baseline phases, as infants were mainly looking at their mother, out of sight of the camera. Infant fussiness was based on separate codings of: (1) duration of infant vocal expressions of fussiness (e.g., crying, whining); (2) intensity of vocal expressions of fussiness; (3); negative facial expressions (i.e., facial tension: stiff mouth, squinted eyes, frowning); and (4) motor activity. For the coding of all variables, each phase of 2 minutes was divided into 10 sec. time intervals. Duration of infant vocal fussiness was coded in number of seconds per interval. All other variables were coded on a 4-point scale ranging from 0 to 3. Higher scores indicated a higher frequency and/or intensity of that behavior. Final scores of each variable were obtained by standardizing and then averaging the scores across time intervals. Cronbach’s alpha of the four infant fussiness variables was .92, indicating a high level of internal consistency. A mean score of infant fussiness per phase was created by averaging the scores on the four variables.

Infant fussiness was coded by eight students, trained by the first author. Twenty percent of videotapes were coded by all students to determine interobserver reliability. Mean interobserver reliability (intraclass correlations; ICC) was .97 (range .88–1.00).

#### Physiological measures

During the six task phases, an ECG for parent and child was taken with a Polar H7 Bluetooth heart rate belt. The inter beat intervals (IBI’s) between R-peaks sent by the Polar were recorded with Vsrrp98 version 10.5 [29], a Windows data acquisition program developed by the technical staff of the Psychology department of the University of Amsterdam. Because the Polar heart rate belt was too large for use with infants, we modified the device so that we could directly connect disposable ECG electrodes (3M Red Dot) to the H7 sensor. At the start of each measurement phase a marker was sent to the recording program Vsrrp98. HR was calculated as the number of IBI’s per minute. HRV was calculated as the root mean square of successive differences in IBI’s (RMSSD), where intervals larger than 133% and smaller than 67% of the preceding IBI were rejected to remove artefacts from the signal. The RMSSD in IBI’s reflects high-frequency variations indicative of parasympathetic activation [30,31]. Mean HR and HRV per minute was calculated per two-minute phase.

### Statistical analyses

Data were normally distributed across all three phases per condition for each outcome variable. Analyses were conducted separately for observed infant fussiness, HR, and HRV. We used two-level (task phase within children) multilevel regression models with restricted maximum likelihood estimation to account for dependency in the outcome variables.

Initially, multilevel models were conducted separately for the smart crib and parent condition, and separately for observed infant fussiness, HR, and HRV. This resulted in six multilevel models. In each multilevel model, we first compared the baseline to supine phase, to investigate whether the supine phase had the desired stressful effect on the infants (manipulation check). Secondly, to test our main hypothesis (i.e., whether infants responded with a CR to a combination of sound, movement, and swaddling), we compared the supine to the soothing phase. Thirdly, we compared the soothing phase to the baseline phase, to investigate whether the infants were calm or calmer during the soothing than during rest, providing information about the extent of relaxation of the infant in the soothing phase. Of note, baseline phases were not included in the multilevel models of observed infant fussiness, as infant fussiness was not coded during the baseline phases. For observed infant fussiness, we thus only directly compared the supine to soothing phase.

To examine whether infant age affected the strength of the CR, we added infant age to these six multilevel models and investigated whether there was a significant interaction between infant age and the effect size for the difference in infant fussiness, HR, and HRV between the supine to the soothing phase.

Lastly, to investigate whether the CR was stronger during the smart crib or parent condition, data of the two conditions were combined and the interaction between condition (smart crib, parent) and phase (supine, soothing) was tested in another multilevel model. Statistical significance was evaluated at α < .05. Data were analyzed with SPSS version 22 [32].

## Results

### Descriptive statistics and preliminary analyses

For two of the 69 participating infants, infant fussiness could not be observed as audio recordings failed. The experiment was ended prematurely for one infant as the mother indicated that her infant was too upset to continue. For one infant, HR and HRV data were not available for the smart crib condition, due to technical problems. Data on observed infant fussiness were thus available for 66 infants, and data on HR and HRV for 67 or 68 infants, depending on the condition.

An immediate Moro response (abduction of the arms, adduction of the arms, and crying) was elicited in 40% of the infants. Infants in which the reflex was elicited were significantly younger (*M* = 10.65 weeks, *SD* = 5.29) than infants in which the reflex was not elicited (*M* = 16.21 weeks, *SD* = 6.86), *t*(66) = 3.57, *p* = .001. Although the Moro was not elicited in all infants, they all displayed a certain level of behavioral fussiness during both supine phases (i.e., on the behavioral observations of duration of infant vocal expressions of fussiness, intensity of vocal expressions of fussiness, negative facial expressions, and motor activity), indicating that our manipulation succeeded.

**Fig 2:**
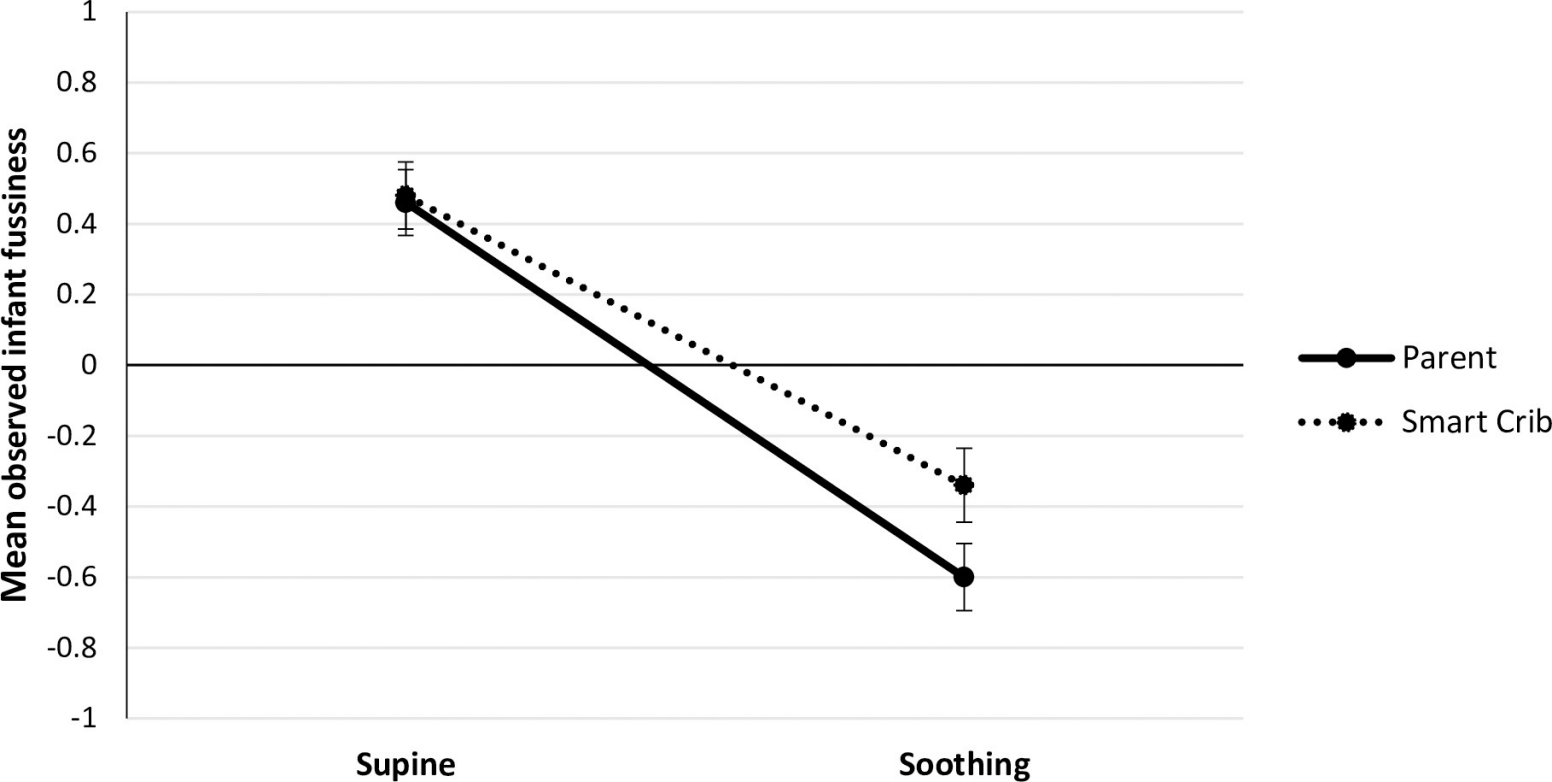
Mean observed infant fussiness during the supine and soothing phases in the parent (*N* = 66) and smart crib condition (*N* = 66). Error bars represent standard errors.

**Fig 3:**
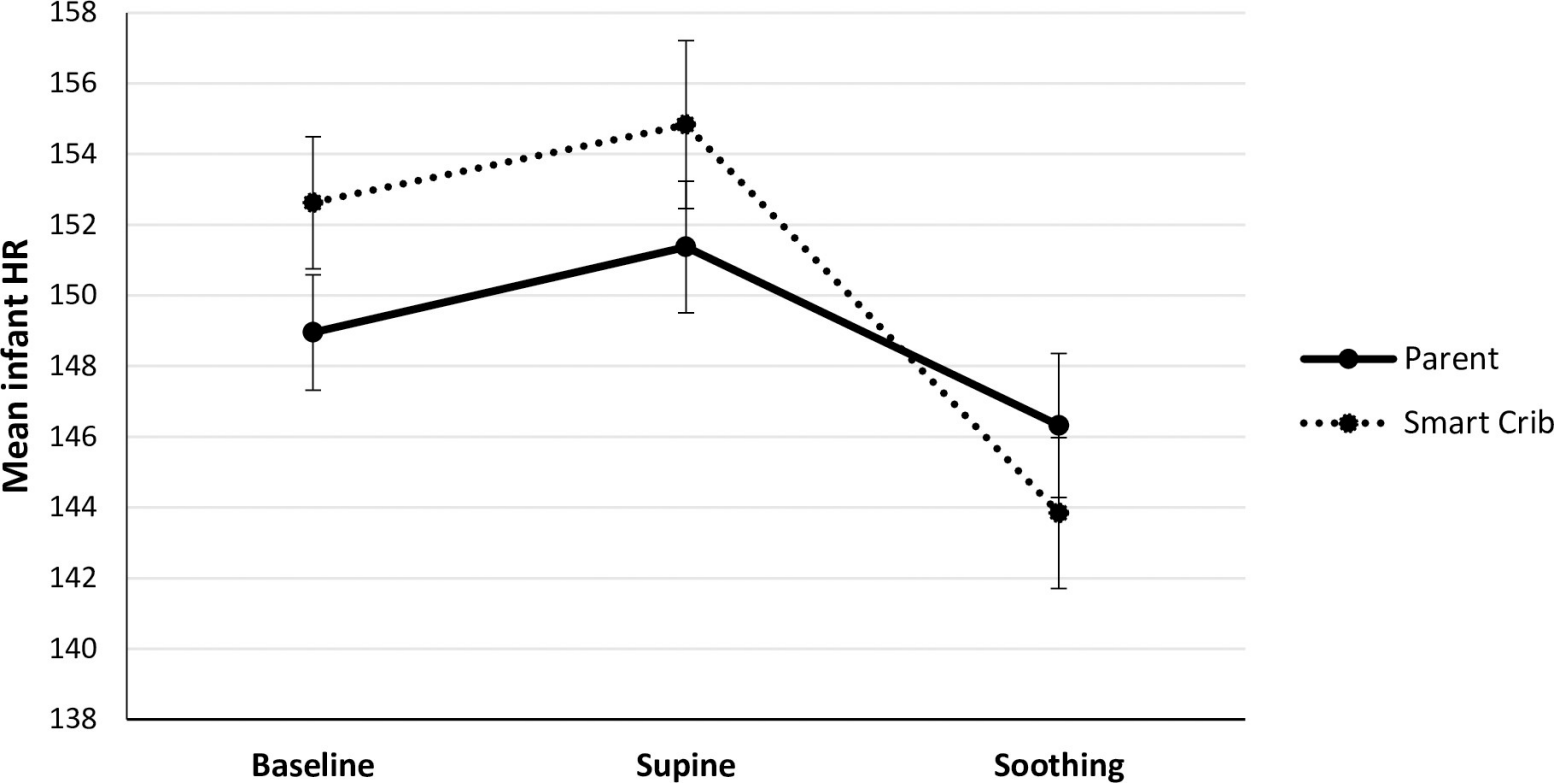
Mean infant HR during the baseline, supine, and soothing phases in the parent (*N* = 68) and smart crib condition (*N* = 67). Error bars represent standard errors.

**Fig 4:**
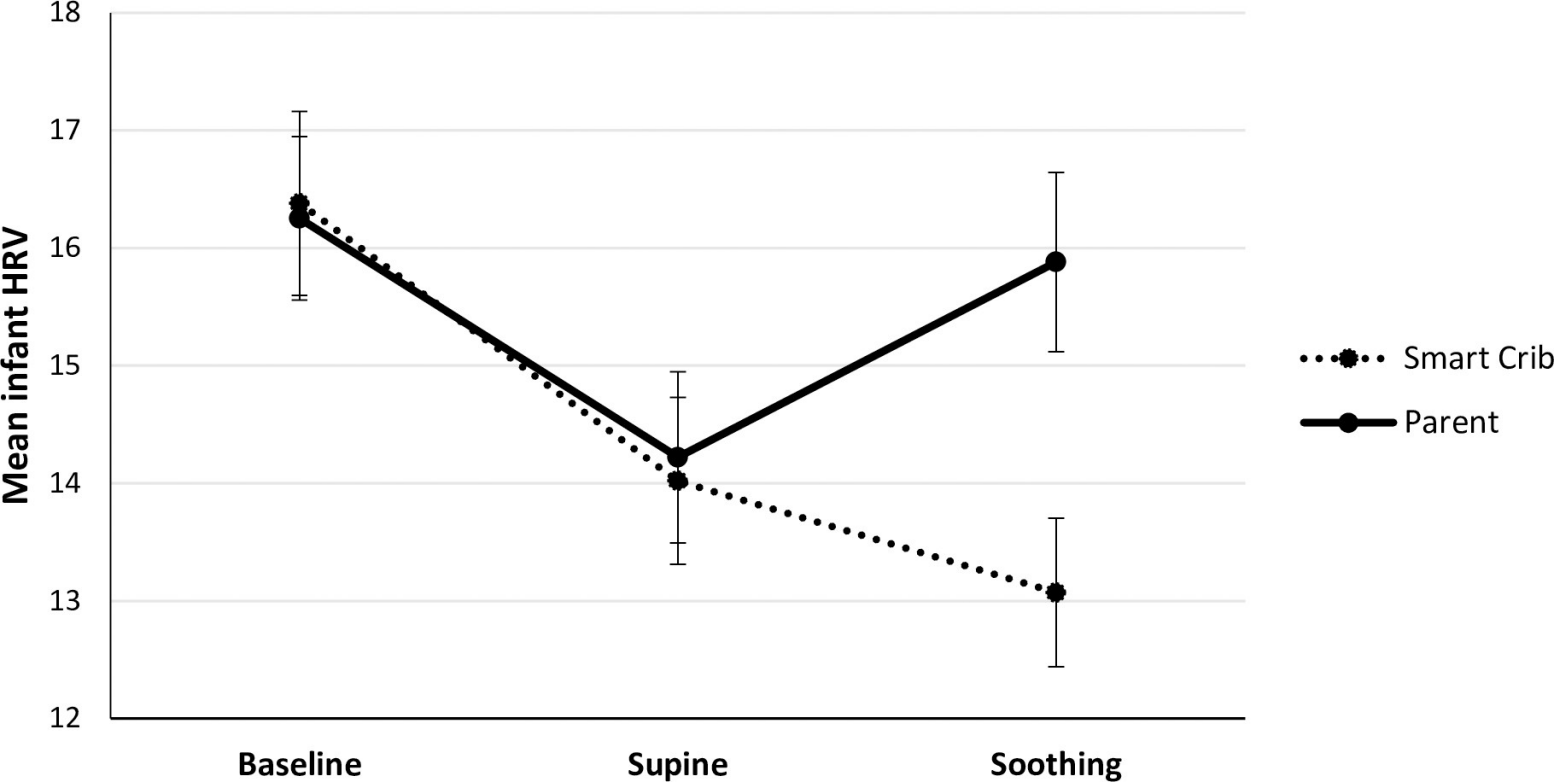
Mean infant HRV during the baseline, supine, and soothing phases in the parent (*N* = 68) and smart crib condition (*N* = 67). Error bars represent standard errors.

Mean levels of observed infant fussiness, infant HR, and infant HRV in the different phases and conditions are presented in Table 1 and Figs 2–4, respectively.

**Table 1:** Mean levels of infant observed infant fussiness, heart rate, and heart rate variability during the three phases (baseline, supine, soothing) and two conditions (parent, smart crib).

	Parent						Smart crib					
	Baseline		Supine		Soothing		Baseline		Supine		Soothing	
Variables	*N*	*M* (*SD*)	*N*	*M* (*SD*)	*N*	*M* (*SD*)	*N*	*M* (*SD*)	*N*	*M* (*SD*)	*N*	*M* (*SD*)
Fussiness	-	-	66	.46 (.76)	66	-.60 (.77)	-	-	66	.48 (.77)	66	-.34 (.85)
HR	68	148.95 (13.48)	68	151.37 (15.35)	68	146.32 (16.79)	67	152.62 (15.26)	67	154.83 (19.47)	67	143.84 (17.44)
HRV	68	16.25 (5.73)	68	14.22 (5.96)	68	15.88 (6.29)	67	16.38 (6.40)	67	14.02 (5.80)	67	13.07 (5.17)

HR, heart rate; HRV, heart rate variability.

### Infants’ calming response

#### Observed infant fussiness

Infant fussiness significantly decreased from supine to parental soothing, *B* = -1.05, *SE* = .11, *t*(65) = -9.44, *p* < .001, and also significantly decreased from supine to soothing by the crib, *B* = -.81, *SE* = .12, *t*(65) = -6.96, *p* < .001.

In the parent condition, the supine-soothing × infant age interaction was significant, *B* = .04, *SE* = .01, *t*(64) = 2.60 *p* = .012. The older the infant, the less strong the decrease in infant fussiness from supine to parental soothing. In the smart crib condition, the supine-soothing × infant age interaction was not significant, *B* = -.00, *SE* = .02, *t*(64) = -.08, *p* = .933.

The decrease in infant fussiness from supine to soothing did not differ significantly between conditions, *B* = -.24, *SE* = .14, *t*(65) = -1.73 *p* = .088, although there was a trend for a stronger CR in response to soothing by the parent than by the smart crib.

#### Infant HR

During the parent condition, infant HR did not significantly differ between the baseline and supine phase, *B* = 2.42, *SE* = 1.73, *t*(67) = 1.40, *p* = .167. Infant HR significantly decreased from supine to parental soothing (*B* = -5.05, *SE* = 2.14, *t*(67) = 2.35, *p* = .021). Infant HR during parental soothing did not significantly differ from HR during baseline, *B* = -2.63, *SE* = 2.27, *t*(67) = -1.16, *p* = .250.

During the smart crib condition, infant HR did not significantly differ between the baseline and supine phase, *B* = 2.20, *SE* = 1.90, *t*(66) = 1.16, *p* = .251. Infant HR significantly decreased from supine to soothing by the crib (*B* = -10.98, *SE* = 2.26, *t*(66) = -4.85, *p* < .001), Moreover, infant HR during soothing by the crib was significantly lower than during baseline, *B* = -8.78, *SE* = 2.32, *t*(66) = -3.79, *p* < .001.

In both the parent and smart crib condition, the supine-soothing × infant age interaction was not significant, *B* = .15, *SE* = .33, *t*(66) = .05, *p* = .964, and *B* = .17, *SE* = .34, *t*(65) = .48, *p* = .630 respectively.

Lastly, we investigated whether the CR was stronger during the parent or smart crib condition. The decrease in HR from supine to soothing was significantly stronger in the smart crib condition than in the parent condition, *B* = -5.96, *SE* = 2.63, *t*(66.65) = -2.27, *p* = .027. Thus, infants responded with a stronger CR in HR to soothing by the smart crib than by the parent.

#### Infant HRV

During the parent condition, infant HRV significantly decreased from baseline to supine, *B* = -1.98, *SE* = .90, *t*(66) = -2.19, *p* = .032. Infant HRV did not significantly differ between the supine and soothing by the parent phase, *B* = 1.61, *SE* = .88, *t*(65.002) = 1.83, *p* = .073, but there was a trend for a higher HRV during parental soothing than during supine. Infant HRV did not differ between the baseline and parental soothing phase, *B* = -.37, *SE* = .90, *t*(66) = -37, *p* < .683.

Regarding HRV during the smart crib condition, infant HRV significantly decreased from baseline to supine, *B* = -2.36, *SE* = .88, *t*(66) = -2.68, *p* = .009. Infant HRV did not significantly differ between the supine and soothing by the crib phases, *B* = -.95, *SE* = .78, *t*(66) = 1.21, *p* = .230. In addition, infant HRV was significantly lower during soothing by the crib than during baseline, *B* = -3.31, *SE* = .87, *t*(66) = -3.78, *p* < .001.

In the parent condition, the supine-soothing × infant age interaction was significant, *B* = -.26, *SE* = .13, *t*(65.52) = -2.00, *p* = .049. The older the infant, the less strong the increase in infant HRV from supine to parental soothing. In the smart crib condition, the supine-soothing × infant age interaction was not significant, *B* = -.17, *SE* = .12, *t*(65) = -1.53, *p* = .131.

Lastly, we investigated whether the CR was stronger in the parent or smart crib condition. Infants responded with a stronger calming response in HRV to soothing by the parent than by the smart crib, *B* = -2.69, *SE* = 1.07, *t*(62.98) = 2.53, *p* = .014.

## Discussion

The purpose of this study was to examine (1) whether swaddling, sound, and movement by means of parental and mechanical soothing elicited a CR in infants; (2) whether infant age affected the strength of the CR; and (3) whether there was a difference in the strength of the CR between parental and mechanical soothing. Infants responded with a CR to a combination of swaddling, sound, and movement during parental and mechanical soothing in terms of infant fussiness and HR. For HRV, there was a trend for parental soothing to elicit a CR in infants, but mechanical soothing did not. Regarding the effects of infant age on the CR, it was found that younger infants responded with a stronger CR (decreased fussiness and increased HRV) to parental soothing, but not to mechanical soothing. Results regarding the differences in the strength of the CR between parental and mechanical soothing were equivocal: for HR, infants’ CR was stronger in the crib than in the parent condition, whereas for HRV, infants’ CR was stronger in the parent condition. For fussiness, infants’ CR tended to be stronger in the parent condition.

Our results suggest occurrence of a CR in response to both soothing techniques. Soothing via swaddling, sound, and movement had a calming effect on observed infant fussiness and on infant physiological activation: infants were observed to be less fussy and had a lower HR when soothed by the parent and the smart crib compared to lying supine. The consistency of the behavioral and HR outcomes suggests that a coordinated CR appears when soothing techniques are used to recover from the distressing situation of lying supine. Infants’ HR during the soothing phases was even lower than during baseline (although not significantly for parental soothing), suggesting that infants’ CR is fundamentally different, and appears more relaxed, than infants’ physiological state during quiet interaction.

Unexpectedly, parental and mechanical soothing did not clearly result in a shift to more parasympathetic activation, as infants’ HRV did not significantly differ between the supine and soothing by the crib and parent phases. Our finding that there was a trend for a higher HRV during parental soothing than during supine was in accordance with expected relaxation during the CR. The slight, but statistically non-significant, decrease during mechanical soothing was opposite to the expected direction. One may question whether lying supine was sufficiently distressing to induce a shift towards more sympathetic and less parasympathetic arousal, allowing us to detect a subsequent CR on the HRV level during the soothing phases. Indeed, the increases in HR during the supine phases were statistically non-significant as compared to baseline. HRV, however, significantly decreased during the supine phases compared to baseline, clearly reflecting the expected parasympathetic withdrawal associated with distress. So, the absence of significant HR increases during lying supine as compared to baseline may be attributable to the slightly arousing nature of the baseline, rather than to the presumed mild stress of lying supine. That is, during the baseline phases, parents were instructed to move as little as possible and to interact only quietly with their infant. The absence of movement and sound might have been mildly stressful for infants. Alternatively, one may suggest to interpret the absence of an increase in HRV during the mechanical soothing as a novelty response. This interpretation is, however, not supported by the observational and HR outcomes, as one would then also expect an increase in HR and less observed behavioral relaxation. Moreover, such a novelty response would not last for two minutes. Taken the previous in consideration, we tend to interpret this HRV finding during mechanical soothing as a spurious outcome, resulting in some inconsistency regarding the outcomes during mechanical soothing.

We expected that the younger the infant, the stronger the CR in response to swaddling, sound, and movement, as swaddling, sound, and movement imitate intra-uterine sensations [10], and as infants’ behavior and physiology gradually shifts from intra-uterine to more extra-uterine regulation during the first months [20–23]. We found some evidence for this hypothesis for parental soothing (for fussiness and HRV), but not for mechanical soothing. That younger infants were not more sensitive to the calming effects of swaddling, sound, and movement by the crib than older infants, might be due to the standardized calming of the crib. The crib always used the same intensity of movement and white noise, whereas swinging and shushing may have varied in terms of intensity during the parental soothing. As a result, even older infants seem to have reacted with an intense CR to soothing by the crib. Thus, our study showed that the CR response is also present in infants over three months of age. As adults are still sensitive to motion [33] and sound [34] to calm down and even fall asleep, the CR in response to sound and motion may rather be an innate and universal characteristic of humans than a unique characteristic of newborns.

The results on the differences in the strength of the CR between parental and mechanical soothing were equivocal. For HRV, infants’ CR was stronger in the parent condition than in the crib condition. For infant fussiness, parental and mechanical soothing did not significantly differ in bringing about a CR in infants, although there was a trend for infants to have a stronger CR in response to parental soothing. On the contrary, infants showed a stronger CR in terms of HR when soothed by the crib than by the parent. Thus, it remains unclear whether parental or mechanical soothing is more effective for calming infants. As humans are inherently social creatures, one might have expected that infants would be more easily soothed by their parent than by a mechanical instrument. Because of their immaturity at birth, infants depend on their caregiving environment to survive and thrive. Indeed, maternal absence or unavailability is associated with infant physiological dysregulation and social withdrawal [35], showing that the mother has an important regulatory function [36]. Our study, however, shows that also mechanical soothing has a calming effect on infants, and may thus be a co-regulator of infants’ behavior and physiology. The crib might be an important help for parents. For instance, parents may quickly become exhausted which poses a risk for parental mental health and sensitive and responsive interaction with the infant, which may result in a vicious cycle of increased infant crying and parental exhaustion [5]. During the night, aiming to prolong infant sleep and to decrease the number of night wakings, the smart crib would not replace the parent, but would replace a conventional crib. Parents would, however, respond to the needs of their baby similarly to when using a normal crib.

The finding that parental soothing using swaddling, shushing, and swinging had a direct calming effect on infants is in concordance with the study of Harrington et al. [17] that showed that the 5S’s of HB as applied by a researcher calmed infants after immunization. Our study shows that HB can also be easily taught to parents. Parents only shortly practiced with the experimenter how to shush and swing their infant. A recent study also showed that excessive crying in infants under 4 months can be significantly diminished by the use of HB [18]. HB thus seems an intervention that can be directly used by parents and professionals to soothe infants.

In the present study, we only investigated the effects of parental and mechanical soothing using swaddling, sound, and movement on infants’ CR, but these sensory stimuli also improve infant sleep. Swaddling has been found to make infants sleep longer [37], white noise promotes falling asleep quicker [38] and promotes waking less during the night [39], and movement accelerates sleep onset and induces deeper sleep [33,40]. The simultaneous use of the 5S’s also seem to boost infant sleep. Paul et al. [41,42] found that in a population of infants at risk for obesity, predominantly breastfed infants receiving responsive care including HB slept more during the night than predominantly breastfed infants not receiving HB. In addition, daily sleep duration also increased for excessively crying infants who were soothed with HB and who were sleeping swaddled and with white noise on [18].

HB might, however, be too challenging if parents are exhausted and sleep deprived. The next step would be therefore to investigate whether the smart crib can also be used to enhance infant sleep and thereby parental sleep. The smart crib might reduce the need of parental interference during the night, and as a result parental night wakings and sleep duration might improve. Especially reducing parental fragmented sleep seems important, since this has a detrimental effect on parental mood [43]. By improving parental sleep via infant soothing and sleep, the vicious cycle between infant crying and sleeping problems on the one hand and parental exhaustion on the other hand may be broken. When rested, parents may be better able to meet the needs of the infant and to react in a more sensitive and responsive way, which can contribute to a better bonding between parent and infant [5]. Results from our first pilot study on the effectiveness of the crib showed that infant crying decreased and infant and maternal sleep improved during intervention with the crib compared to baseline [44].

The results of our study should be interpreted with several limitations in mind. Firstly, the results of our study must be interpreted carefully, as we did not correct for multiple testing. Relatedly, some of the non-significant findings might be due to power issues, given the relatively low number of parents and infants in our study sample. Replication of our findings with larger samples is necessary.

Secondly, the level of elicited distress was only mild. On the one hand, this could have resulted in an underestimation of potential effects of our soothing methods on behavioral and physiological characteristics of distress. On the other hand, it remains unknown whether a CR can still be evoked using swaddling, sound, and movement when infants experience more intense distress. Some support for the calming effects of swaddling, sound, and motion in response to a more intense stressor comes, however, from the study of Harrington et al. [17] that showed that the 5S’s calm infants after immunization, which is a painful procedure for infants. Nevertheless, studies investigating the CR when inducing a higher level of infant distress are needed.

Thirdly, infant fussiness could not be observed in the baseline phases, because most infants were looking at their parents and cameras could not capture infants’ faces. During the pilot testing of the study, we tried to let all infants sit still at their parents lap with their back against the chest of the parent so that infants’ faces could be fully filmed, but this position made some infants fussy. As the baseline phase should represent a calm state, parents could ultimately choose themselves how they would like to sit with their infant, as long as parent and child moved as little as possible.

Fourthly, we did not determine the effectiveness of the separate sensory stimuli as swaddling, sound, and movement were applied simultaneously. Although there is already evidence for a calming effect of each of the three forms of stimulation, experimental studies might be useful for disentangling which or which combination of the 5S’s is most effective for eliciting the CR of infants. For some infants, swaddling may be enough, whereas some infants may be more sensitive to movement, and other infants respond more to sound, or need a combination of (some of) the stimuli.

Fifthly, most participating parents were female and from a high social economic background, and most infants were born term, possibly limiting the generalizability of our findings. In addition, parents and infants were from a community sample, and not specifically at high risk for excessive crying. As the CR is assumed to be a universal infant response [10], we expect a CR can be elicited in all infants using swaddling, sound, and movement, also in excessively crying infants. Results from our studies indeed show that swaddling, sound, and movement has a calming effect on excessively crying infants, when soothed by the parent with HB [18], and with the smart crib [44]. Future studies should compare the elicitation of the CR with parental versus mechanical soothing in excessively crying infants. Another interesting population to study is preterm born children, who often have difficulty with sensory modulation (i.e., regulating the intensity of responses to sensory stimuli), which results in greater than typical irritability to sensory stimulation [45]. Preterm infants, however, may be especially responsive to the sensations that infants experienced in the womb because of their immaturity. It has been suggested that behavioral state regulation in preterm born infants may be impaired due to lack of intra-uterine entrainment [46]. Preterm infants exposed to cycled light cried less and were less fussy at 5 and 11 weeks corrected age than preterm infants exposed to dimmed light [46,47]. However, preterm infants’ pattern of crying after their due date resembled that of term-born infants [48]. This may indicate that preterm infants also need intra-uterine stimuli corresponding to the prolonged intra-uterine effect or the period before the first biobehavioral shift [22,23]. This might provide opportunities for early interventions directed at the infant’s sensory processing as these may help to enhance the self-regulatory capacities of the preterm infant.

To conclude, parental and mechanical soothing using swaddling, sound, and movement promptly induced a CR in infants. This indicates that newborns are very sensitive to these intra-uterine stimuli. This finding might have important clinical implications for the soothing of fussy and crying infants and specifically in the context of parental exhaustion. Future studies are needed to investigate the effects of parental versus mechanical soothing in the home setting.

## Supporting information

S1 File Dataset infant crying and the calming response. (SAV)

S1 Table Codebook dataset. (DOCX)
