# Supplementary material for: Infant crying and the calming response: Parental versus mechanical soothing using swaddling, sound, and movement
Source: PLoS One. 2019 Apr 24;14(4):e0214548. doi: 10.1371/journal.pone.0214548 (PMC6481793; doi:10.1371/journal.pone.0214548)
Supplement: S1 Table — (DOCX) [file pone.0214548.s002.docx]

| **Variable Information** | | | | | | | | | |
| --- | --- | --- | --- | --- | --- | --- | --- | --- | --- |
| Variable | Position | Label | Measurement Level | Role | Column Width | Alignment | Print Format | Write Format | Missing Values |
| ID | 1 | ID | Scale | Input | 5 | Right | F40 | F40 |  |
| Gender_c | 2 | Child gender | Scale | Input | 5 | Right | F40 | F40 |  |
| Gender_p | 3 | Parent gender | Scale | Input | 5 | Right | F40 | F40 |  |
| Living_status_p | 4 | Living status parent | Scale | Input | 16 | Right | F40 | F40 |  |
| Education_p | 5 | Parental education | Scale | Input | 34 | Right | F40 | F40 |  |
| Birth_country_p | 6 | Birth country parent | Nominal | Input | 18 | Left | A30 | A30 |  |
| Gest_age_c | 7 | Gestational age child in weeks | Scale | Input | 10 | Right | F8.2 | F8.2 | 999,00 |
| Corr_age_c | 8 | Corrected age child in weeks | Scale | Input | 18 | Right | F8.2 | F8.2 | 999,00 |
| Age_p | 9 | Age parent | Scale | Input | 12 | Right | F8.2 | F8.2 | 999,00 |
| Condition_first | 10 | First condition during experiment | Nominal | Input | 10 | Right | F8 | F8 | 999 |
| HR_baseline_parent_c | 11 | HR baseline before parent soothing | Scale | Input | 20 | Right | F8.2 | F8.2 | 999,00 |
| HR_supine_parent_c | 12 | HR supine before parent soothing | Scale | Input | 15 | Right | F8.2 | F8.2 | 999,00 |
| HR_parent_c | 13 | HR parent soothing | Scale | Input | 12 | Right | F8.2 | F8.2 | 999,00 |
| HR_baseline_crib_c | 14 | HR baseline before crib soothing | Scale | Input | 20 | Right | F8.2 | F8.2 | 999,00 |
| HR_supine_crib_c | 15 | HR supine before crib soothing | Scale | Input | 15 | Right | F8.2 | F8.2 | 999,00 |
| HR_crib_c | 16 | HR crib soothing | Scale | Input | 12 | Right | F8.2 | F8.2 | 999,00 |
| HRV_baseline_parent_c | 17 | HRV baseline before parent soothing | Scale | Input | 21 | Right | F8.2 | F8.2 | 999,00 |
| HRV_supine_parent_c | 18 | HRV supine before parent soothing | Scale | Input | 16 | Right | F8.2 | F8.2 | 999,00 |
| HRV_parent_c | 19 | HRV parent soothing | Scale | Input | 13 | Right | F8.2 | F8.2 | 999,00 |
| HRV_baseline_crib_c | 20 | HRV baseline before crib soothing | Scale | Input | 21 | Right | F8.2 | F8.2 | 999,00 |
| HRV_supine_crib_c | 21 | HRV supine before crib soothing | Scale | Input | 16 | Right | F8.2 | F8.2 | 999,00 |
| HRV_crib_c | 22 | HRV crib soothing | Scale | Input | 13 | Right | F8.2 | F8.2 | 999,00 |
| Fussiness_supine_crib_c | 23 | Infant fusiness supine before crib soothing | Scale | Input | 17 | Right | F8.2 | F8.2 | 999,00 |
| Fussiness_crib_c | 24 | Infant fussiness crib | Scale | Input | 14 | Right | F8.2 | F8.2 | 999,00 |
| Fussiness_supine_parent_c | 25 | Infant fussiness supine before parental soothing | Scale | Input | 17 | Right | F8.2 | F8.2 | 999,00 |
| Fussiness_parent_c | 26 | Infant fussiness parent | Scale | Input | 14 | Right | F8.2 | F8.2 | 999,00 |
| Variables in the working file | | | | | | | | | |

| **Variable Values** | | |
| --- | --- | --- |
| Value | | Label |
| Gender_c | 1 | Male |
|  | 2 | Female |
| Gender_p | 1 | Male |
|  | 2 | Female |
| Living_status_p | 1 | Living together with partner and child(ren) |
|  | 2 | Living apart with child(ren) |
|  | 3 | Other, specify |
| Education_p | 1 | Elementary school/primary school |
|  | 2 | Junior general secondary education (vmbo/mavo) |
|  | 3 | Senior general secondary education (havo) |
|  | 4 | University preparatory education (vwo) |
|  | 5 | Senior secondary vocational education (mbo) |
|  | 6 | Higher professional education (hbo) |
|  | 7 | Academic higher education (university) |
|  | 8 | Different, specify |
| Condition_first | 1 | Parent first |
|  | 2 | Crib first |
